# Choosing an Optimal Solvent Is Crucial for Obtaining Cell-Penetrating Peptide Nanoparticles with Desired Properties and High Activity in Nucleic Acid Delivery

**DOI:** 10.3390/pharmaceutics15020396

**Published:** 2023-01-24

**Authors:** Abhijit Biswas, Maria Maloverjan, Kärt Padari, Aare Abroi, Margus Rätsep, Sebastian K. T. S. Wärmländer, Jüri Jarvet, Astrid Gräslund, Vambola Kisand, Rünno Lõhmus, Margus Pooga

**Affiliations:** 1 Institute of Technology, University of Tartu, 1 Nooruse Street, 50411 Tartu, Estonia; 2 Institute of Molecular and Cell Biology, University of Tartu, 23b Riia Street, 51010 Tartu, Estonia; 3 Institute of Physics, University of Tartu, 1 Wilhelm Ostwaldi, 51014 Tartu, Estonia; 4 Chemistry Section, Stockholm University, Arrhenius Laboratories, SE-10691 Stockholm, Sweden; 5 CellPept Sweden AB, Kvarngatan 10B, SE-11847 Stockholm, Sweden

**Keywords:** cell-penetrating peptides, solvent, nanoparticle formation, nucleic acid delivery

## Abstract

Cell-penetrating peptides (CPPs) are highly promising transfection agents that can deliver various compounds into living cells, including nucleic acids (NAs). Positively charged CPPs can form non-covalent complexes with negatively charged NAs, enabling simple and time-efficient nanoparticle preparation. However, as CPPs have substantially different chemical and physical properties, their complexation with the cargo and characteristics of the resulting nanoparticles largely depends on the properties of the surrounding environment, i.e., solution. Here, we show that the solvent used for the initial dissolving of a CPP determines the properties of the resulting CPP particles formed in an aqueous solution, including the activity and toxicity of the CPP–NA complexes. Using different biophysical methods such as dynamic light scattering (DLS), atomic force microscopy (AFM), transmission and scanning electron microscopy (TEM and SEM), we show that PepFect14 (PF14), a cationic amphipathic CPP, forms spherical particles of uniform size when dissolved in organic solvents, such as ethanol and DMSO. Water-dissolved PF14, however, tends to form micelles and non-uniform aggregates. When dissolved in organic solvents, PF14 retains its α-helical conformation and biological activity in cell culture conditions without any increase in cytotoxicity. Altogether, our results indicate that by using a solvent that matches the chemical nature of the CPP, the properties of the peptide–cargo particles can be tuned in the desired way. This can be of critical importance for in vivo applications, where CPP particles that are too large, non-uniform, or prone to aggregation may induce severe consequences.

## 1. Introduction

Gene therapy is an explosively developing field of nanomedicine and holds promise for treating various diseases that are undruggable today [1,2,3,4]. Although viral delivery systems have historically been highly important and widely applicable to the stable expression of various proteins, they have some inherent disadvantages, such as immunogenicity and potential carcinogenicity [5,6,7,8]. However, the development of non-viral delivery systems for nucleic acids (NAs) is challenging because of their limited ability to cross the plasma membrane. Low cellular uptake and inefficient endosomal escape are the main barriers to the widespread application of NAs since only a small proportion, usually, less than 0.1% of the cargo, reaches the cytoplasm and can be biologically active [9]. Thus, the development of safe and efficient delivery vehicles is necessary for the wide clinical application of therapeutic NAs [10].

In recent decades, multiple non-viral delivery systems have been developed, including physical approaches, such as electroporation and microinjection, and chemical delivery vectors, such as lipids, polymers, and peptides [11]. Among other delivery vehicles, cell-penetrating peptides (CPPs) have emerged as potential tools for the cellular delivery of multiple types of cargo molecules [12,13,14,15,16]. CPPs are peptides that are less than 30 amino acids long, which are typically cationic and/or amphipathic and can be associated with their cargo molecules both via covalent bonding and by non-covalent complex formation [17,18,19,20]. They have been shown to mediate the cellular uptake of biologically relevant cargo molecules of different sizes and chemistry, including small interfering RNA (siRNA) [21,22], antisense oligonucleotides (ASOs) [23,24], plasmid DNA (pDNA) [25,26], messenger RNA (mRNA) [27], and proteins [28], both in vitro and in vivo.

To become a candidate for clinical use, a pharmaceutical formulation must have a well-defined molecular composition, appropriate size, and suitable structural properties [29]. For the association of cationic CPPs with negatively charged molecules, such as NAs, simple mixing is often sufficient, as electrostatic interactions enable the rapid formation of stable complexes that assemble into nanoparticles. This approach is highly attractive due to its simplicity and low time consumption. However, the resulting complexes/particles tend to be heterogeneous, which is characteristic of the majority of self-assembling systems. Uneven physicochemical characteristics of the particle population, especially aggregates, can cause toxicity and affect the biodistribution of the cargo [29,30]. Thus, it is essential to prepare well-characterised, highly homogeneous nanoparticle formulations, which is especially important for in vivo applications [29].

One of the key factors that define the properties of the resulting particles is the solvent used for their preparation [31], as well as for the initial dissolution of the components. The solubility of a compound depends on its relationship with a solvent. Polar solvents such as water, methanol (MeOH), ethanol (EtOH), and isopropanol (iPrOH) dissolve polar and ionic compounds, while the solubility of non-polar compounds, such as lipids or aromatic compounds, is usually poor in polar solvents. However, the addition of semi-polar solvents, such as dimethyl sulfoxide (DMSO), dimethylformamide (DMF), etc., to polar solvents can enhance the solubility of non-polar compounds in polar solvents and stabilise solutions of amphiphiles, such as fatty-acid-modified CPPs.

The self-assembly of biologically active molecules into functionally active complexes is an important phenomenon for imparting their activity in biological systems [31,32]. Cosolvents that help to solubilise and later mask hydrophobic regions play a vital role in the self-assembly of biomacromolecules in aqueous solutions in in vitro assays [33,34,35]. In natural environments, e.g., in the cell cytosol, there are many cosolvents, i.e., osmolytes, such as the denaturant urea and trimethylamine N-oxide (TMAO) [36,37], which facilitate the self-assembly of protein complexes with other proteins, NAs, or lipids, and regulate their functioning by building up hydrogen bonds with water [33,37]. However, the identification of a particular cosolvent that enables the generation of functional biologically active soft materials with desired properties is not a straightforward or easy task.

In this work, we dissolved CPPs in different solvents and examined their effect on the CPP-mediated cellular delivery of RNA. We mainly analysed the ability of differently dissolved PepFect14 (PF14) peptides to deliver splice-correcting oligonucleotide (SCO) and siRNA into mammalian cell lines. In addition, we characterised PF14 complexes with SCO that assemble into colloidal nanoparticles using a battery of biophysical in vitro methods. The used prototypic fatty acid-modified CPP, i.e., PF14, is an amphipathic stearylated peptide from the PepFect family that was first synthesised by Ezzat et al. in 2011 [38]. It carries a positive charge of +5 that enables the formation of non-covalent complexes with NAs, which are internalised by cells via scavenger receptor-mediated endocytosis [38,39,40]. Peptides from the PepFect family have shown high efficiency in the delivery of SCO [39,41], siRNA [42], miRNA [43,44,45,46], and other types of NA molecules into mammalian cells both in vitro and in vivo and have been extensively studied by our group for several years [39,40,47,48].

## 2. Materials and Methods

### 2.1. Materials

SCO-705 (5′-CCUCUUACCUCAGUUACA-3′), SCO-654 (5′-GCUAUUACCUAAACCCAG-3′), and Cy5-SCO-654, all with a phosphorothioate backbone and 2′OMe modification, were purchased from Metabion (Planegg, Germany). The siRNA targeting luciferase (5′-GGACGAGGACGAGCACUUCTT-3′) and the negative control siRNA (5′-AGGUAGUGUAAUCGCCUUGTT-3′) were obtained from Microsynth (Balgach, Switzerland). The following CPPs were used: PF14, hPep3, and CADY (see Table S1). PF14 was obtained from PepMic (Suzhou, China), CADY was kindly provided by G. Divita and S. Deshayes, Montpellier CNRS, and hPep3 by T. Lehto, ITUT. For all peptides, 1 mM stock solutions were prepared and stored at −20 °C. Lipofectamine RNAiMAX Reagent was obtained from Invitrogen (Waltham, MA, USA). The used organic solvents were of p.a. grade with low water content (>99.5%): EtOH (Chem-Lab, Zedelgem, Belgium), 2-propanol and 2-butanol (Riedel-de Häen, Honeywell, Wabash, IN, USA), DMSO (AppliChem, Darmstadt, Germany), and THF (Lachner, Eurasburg, Germany). Trimethylene carbonate, i.e., 1,3-dioxan-2-one, was sourced from Apollo (Stockport, UK), and ethylene carbonate and glycerol carbonate were sourced from Acros Organics (Geel, Belgium). Other reagents, such as CaCl_2_, MgCl_2_, MeOH, DMF, etc., were obtained from Merck-Sigma-Aldrich (Darmstadt, Germany) if not stated otherwise. The cell culture lysis solution contained 1% (*v*:*v*) Triton X-100, 50 mM of Tris (pH 7.5), 150 mM of NaCl, and 1 mM of EDTA. Ready-to-use Cell Proliferation Colorimetric Reagent, WST-8, was purchased from BioVision (Milpitas, CA, USA). The luciferase activity measurement protocol was based on Rocha et al., 2016 [49] and Helmfors et al., 2015 [50]. The silicon wafers used for scanning electron microscopy and atomic force microscopy were obtained from Micro to Nano (Haarlem, The Netherlands).

### 2.2. PF14-SCO Nanoparticle Formation

For the splice-correction assay, we prepared CPP-SCO nanoparticles by mixing them at a 5:1 molar ratio (MR) in Milli-Q (MQ) water in 1/10th of the final volume [51]. After mixing by pipetting, the solutions were incubated at room temperature (RT) for 15 min. Then, if needed, divalent metal ions in the form of CaCl_2_ or MgCl_2_ (at a final concentration of 3 mM) were added, and the solutions were incubated for 15 min more. Finally, the particle-containing solutions were diluted 10× with pre-warmed cell culture media and applied to the cells. For the gene silencing assay, the CPPs were complexed with siRNA at a 34:1 MR, following the same protocol as described for CPP-SCO complexes. In all experiments, a cell culture medium containing 10% (*v*:*v*) of MQ water was used as a negative control solution. In the gene silencing experiments, the CPP-siRNA particles that contained non-targeting siRNA (siNEG) were used as a negative control, and the particles prepared using siRNA and Lipofectamine RNAiMAX (Invitrogen) were used as a positive control.

### 2.3. Dynamic Light Scattering Analysis

The hydrodynamic diameters and zeta potentials of the nanoparticles were analysed using a Zetasizer Nano ZSP instrument (Malvern Panalytical, Malvern, UK). To measure the size of the PF14 particles, the 1 mM stock was diluted 100-fold in MQ water at RT and allowed to equilibrate for at least 30 min. The CPP-SCO nanoparticle solutions were prepared as described previously at 5:1 MR with or without the addition of CaCl_2_ or MgCl_2_. After 15 min of incubation at RT, the solutions were diluted 10× with MQ water to reach the final volume of 1 mL and again incubated at RT for 15 min before measurement. Three to five consecutive measurements were performed depending on the homogeneity of the particle sizes.

### 2.4. Cell Culture

For the splice-correction assay, two human beta-thalassemia reporter cell lines, i.e., HeLa pLuc 705 [52] and HeLa EGFP 654 [53,54], were used. For the gene-silencing assay, a luciferase-expressing cell line, i.e., U87 MG-Luc2 [55], was used. The cells were cultured at 37 °C in Dulbecco’s Modified Eagle Medium (DMEM) (Corning, Corning, NY, USA) containing 4.5 g/L of glucose and supplemented with 10% (*v*:*v*) of fetal bovine serum (FBS) and 1% (*v*:*v*) of a penicillin–streptomycin solution (100 U/mL penicillin and 100 g/mL streptomycin). The cells were grown on 6 cm cell culture dishes (Corning) in a humid atmosphere that contained 5% CO_2_. The cells were split every second day. To detatch the cells, trypsin-EDTA solution (Corning) was used. The cells were washed with Dulbecco’s Phosphate-Buffered Saline (DPBS) without calcium and magnesium (Corning). To conduct the experiments, the cells were plated on 96-well (Sarstedt, Nümbrecht, Germany) or 24-well (CytoOne, Hamburg, Germany) plates. All of the operations using cells were performed in a sterile cell culture hood.

### 2.5. Luminescence Measurement

For the luminescence measurements, HeLa pLuc 705 and U87 MG-Luc2 cells were plated on a 96-well plate at 10,000 cells/well and 8000 cells/well, respectively, 24 h before transfection and incubated with nanoparticles, as described above. After 24 h (for HeLa pLuc 705 cells) or 48 h (for U87 MG-Luc2 cells) of incubation with nanoparticle-containing solutions, the cells were washed with PBS and lysed by adding 20 µL of cell culture lysis buffer [56] containing 1% Triton-X100 and freezing the solutions at −25 °C. After thawing the solutions at room temperature, 70 µL of the luciferase substrate solution [56] per well was added, and the solutions were mixed and transferred to a white 96-well plate (Greiner Bio-One, Kremsmünster, Austria). Finally, the luminescence intensity was measured with an Infinite M200 PRO microplate reader (Tecan, Männedorf, Switzerland), using Magellan 7 software.

### 2.6. Cytotoxicity Assay

The effects of the peptides and peptide-containing nanoparticles on the viability of the cells were measured using the WST-8 assay (APExBIO, Boston, MA, USA). The cells were seeded on a 96-well plate the day before transfection. On the next day, the cells were transfected as described above. After 24 h (for the SCO assay) or 48 h (for the siRNA assay) of incubation, the media was replaced with fresh media that contained 10% of WST-8 solution, and the plate was incubated at 37 °C for 3 h before taking the measurement of the absorbance. The absorbance of the solutions was recorded at a 450 nm wavelength with a reference wavelength of 600 nm using an Infinite M200 PRO microplate reader (Tecan). The absorbance of the wells without cells but with medium and WST-8 was subtracted from all other values. The absorbance of the untreated cells was taken for 100% viability. As a positive control, the cells were incubated with 10% DMSO solution in the medium.

### 2.7. Confocal Microscopy

For the confocal microscopy analysis, HeLa EGFP 654 cells were plated in a 24-well plate at 50,000 cells/well on cover glasses with a diameter of 12 mm (Menzel-Gläser, Braunschweig, Germany) 24 h before transfection. On the next day, the medium was replaced with a nanoparticle-containing medium. The nanoparticles were prepared as described above. Here, we used SCO-654:Cy5-SCO-654 at a 9:1 ratio (180 nM SCO-654 and 20 nM Cy5-SCO-654), 1 µM PF14 dissolved in EtOH90/DMSO10/TMC0.4, and 3 mM CaCl_2_. The cells were incubated with the solutions for 24 h at 37 °C. On the next day, the cells were washed with PBS and fixed by incubation with 3% paraformaldehyde (PFA) for 30 min at RT. The cell nuclei were stained with 4,6-diamidino-2-phenylindole (DAPI) 0.5 µg/mL solution in PBS. The cells were washed with PBS and mounted to glass slides in 30% glycerol. The specimens were analysed using an Olympus FluoView FV1000 (Olympus, Tokyo, Japan) confocal microscope. For each solution, two specimens were prepared, and at least three images per specimen were obtained (with circa 10 layers per image with a step size of 1.2 µm). Cy5 (λ_ex/em_ = 633/666 nm), EGFP (λ_ex/em_ = 488/530 nm), and DAPI (λ_ex/em_ = 405/470 nm) signals were analysed, and a 60× objective with water immersion was used.

### 2.8. Flow Cytometry

For the flow cytometry, HeLa EGFP 654 cells were plated in a 96-well plate 24 h prior to transfection. On the next day, the cells were transfected with nanoparticles prepared of 100 nM SCO-654, 10 nM Cy5-SCO-654, 500 nM PF14, and 3 mM CaCl_2_ or MgCl_2_, as described above. The cells were incubated with the nanoparticles for 24 h at 37 °C and then washed with PBS, detached with a trypsin-EDTA solution, and resuspended in PBS. The cell nuclei were stained with 0.5 µg/mL DAPI for the gating out of dead cells. Next, flow cytometry of the cell suspensions was performed with an Attune NxT flow cytometer (Thermo Fisher Scientific, Waltham, MA, USA), using DAPI (λ_ex/em_ = 405/470 nm), Cy5 (λ_ex/em_ = 633/666 nm), and EGFP (λ_ex/em_ = 488/530 nm) channels. For each sample, 10,000 events were analysed.

### 2.9. Transmission Electron Microscopy (TEM)

For TEM imaging, stock solutions of PF14 were diluted 10-fold in MQ water and drop-casted on carbon-coated copper grids. The specimens were air-dried for 5 min and stained with 2% aqueous uranyl acetate solution. PF14-SCO particles were prepared as described above using a 10:1 MR (PF14:SCO). Next, the samples were air dried and analysed with a Tecnai G2 Spirit transmission electron microscope (FEI, Hillsboro, OR, USA) operating at a 120 kV accelerating voltage, and images were captured using an Orius SC1000 camera and analysed with ImageJ software (NIH, Bethesda, MD, USA).

### 2.10. Atomic Force Microscopy (AFM)

For the AFM analysis of the nanoparticles, PF14-SCO complexes were prepared at a 10:1 MR (PF14:SCO; 1 µM SCO) in MQ water with or without the addition of 3 mM CaCl_2_ or MgCl_2_, which was conducted in the same way as the nanoparticles prepared for the cell-based assay. Then, 5 µL of the solution was spotted on a silicon wafer and air-dried at ambient temperature. The samples were examined with a Veeco Dimension Edge AFM unit (Veeco Instruments Inc., Plainview, NY, USA), and the data were analysed using Gwyddion software (Czech Metrology Institute, Jihlava, Czech Republic).

### 2.11. Scanning Electron Microscopy (SEM)

To analyse the morphology of both the bare peptide and the peptide–SCO complexes with or without the addition of CaCl_2_ or MgCl_2_, we prepared the complexes in the same way as for AFM. The samples were analysed using a NOVA scanning electron microscope (FEI, Hillsboro, OR, USA), and the images were analysed using ImageJ software.

### 2.12. Circular Dichroism Spectroscory (CD)

The circular dichroism spectra of the PF14 peptide dissolved in different solvents were recorded in a Chirascan-plus CD-spectrometer (Applied Photophysics, Leatherhead, UK). The peptides were diluted in MQ water to reach a concentration in the range of 60 to 80 µM for the measurement. The spectra were recorded between 195 nm and 250 nm using a quartz cuvette with a path length of 1 mm. The spectra were smoothed and plotted using GraphPad Prism 8.0 (GraphPad Software, Inc., San Diego, CA, USA).

### 2.13. Energy-Dispersive X-ray Spectrometry (EDX)

The qualitative and quantitative elemental analyses of the peptide–oligonucleotide–metal ion-containing nanoparticles were examined using EDX analysis. The nanoparticle specimens were prepared as described above for SEM and AFM. The EDX spectra were recorded in an Oxford Instruments X-Max system coupled to a Carl Zeiss SUPRA 55VP FE-SEM instrument (Zeiss AG, Oberkochen, Germany). INCA software was used to evaluate the data with respect to the carbon content of the samples, i.e., all atomic percentages were calculated as the % of carbon atoms.

### 2.14. Statistical Analysis

All of the statistical analyses were carried out using GraphPad Prism 5.0 software. Each dataset represents mean ± SD. The statistical significance of the differences between the datasets was analysed using one-way ANOVA with post-hoc Tukey’s or Dunnett’s test at a significance level of 0.05.

## 3. Results and Discussion

### 3.1. PF14 Dissolved in Polar Organic Solvents Assembles into Nanoparticles in Water

The dissolution of PF14 or its analogues, where the stearyl moiety is replaced with different saturated fatty acids in water, leads to the formation of long micelles and/or aggregates of different sizes [57]. On the other hand, the condensation of NA molecules of different sizes by complex formation with PepFect (PF) and NickFect (NF) peptides in water leads to the formation of nanoparticles with a diameter in the range of 50 to 100 nm [58]. However, in addition to the nanoparticles, the PF14-NA solution also contains very large particles/aggregates, especially when complexes are formed at high concentrations of the constituents [29]. Large aggregates and conglomerates of nanoparticles are considered to strongly contribute to the toxicity and immunogenicity of nanoparticle formulations [59]. We presume that aggregates of PFs that form when the peptide is dissolved in water do not completely dissociate during complex formation with NAs, and some of these produce very large particles, leading to unwanted effects that mostly manifest in in vivo experiments. We hypothesised that harnessing a suitable organic solvent for dissolving PF14 could reduce the formation of large aggregates in aqueous solutions since the peptide then retains its microenvironment [36]. This, in turn, could also suppress the formation of aggregates in PF14-NA nanoparticle formulations. In principle, if we can reduce the toxicity and other side effects of CPP-NA nanoparticles in vivo, we could even accept a small loss in activity if needed.

However, choosing a proper solvent to dissolve a CPP that is later used for the preparation of nanoparticles is a challenging task. The Food and Drug Administration of the USA (FDA) divides solvents into four classes [60] according to their safety for patients and environmental impacts. We excluded the application of class I solvents, which are defined as highly toxic and include substances such as benzene and dichloroethene. Class II solvents display lower but substantial toxicity and can be included in pharmaceutical products at very limited concentrations, and their examples include MeOH, toluene, tetrahydrofuran (THF), etc. For this study, class III solvents were primarily used, which are considered safe for humans when applied in amounts not exceeding 50 mg per day (for some of them, this amount can be higher). Examples of such solvents include EtOH, acetic acid, and DMSO.

First, we dissolved PF14 in the series of organic solvents commonly used at moderate concentrations in experiments with cultured cells and in vivo, such as EtOH, DMSO, iPrOH, and MeOH. All of these solvents dissolved PF14 quickly and completely up to a 10 mM concentration, which was the highest concentration tried. Water, in contrast, dissolved PF14 more slowly, even at a 1 mM concentration, which is the most commonly used concentration of stock solutions of this type of peptide.

**Figure 1:**
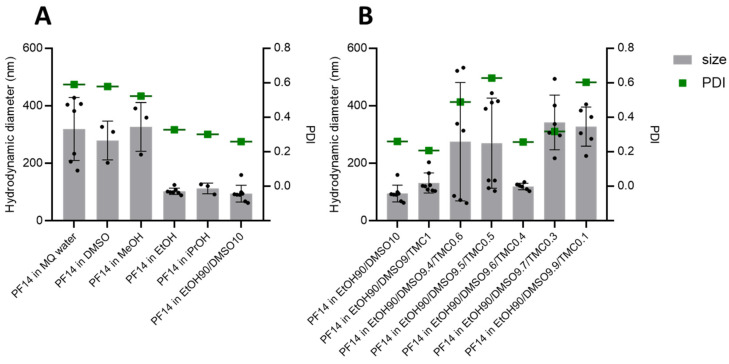
Hydrodynamic diameter and polydispersity indexes (PDI) of particles formed by differently dissolved PF14. (**A**) Particles formed by PF14 dissolved in MQ water, alcohols, and the mixture of EtOH and DMSO. (**B**) Particles formed by PF14 dissolved in a mixture of EtOH and DMSO with addition of TMC at different percentages. The 1 mM PF14 stock solutions in different solvent mixtures were diluted 10× with MQ water and incubated at room temperature for 30 min before measurement with Malvern Zetasizer Nano. Each dataset mean ± SD of at least three measurements with individual measurements indicated as black dots. The polydispersity indexes are presented as the average of at least three independent measurements. All proportions are given as *v*:*v*.

Next, we diluted 1 mM PF14 stock solutions into MQ water to the 10 µM concentration that is commonly used for the preparation of its complexes with NAs to be delivered into cells. Analysis of the size and homogeneity of the nanoparticles formed by PF14 dissolved in different solvents by dynamic light scattering (DLS) showed a very high variation between the nanoparticles prepared with different solvents (Figure 1). The PF14 stocks in EtOH and iPrOH produced particles with diameters ranging from 100 to 200 nm and a rather fair homogeneity, whereas the stocks in water, DMSO, and MeOH produced larger particles/aggregates that were around 300 nm large and had high polydispersity indexes (Figure 1). The numerical values of all particle diameters and polydispersity indexes can be found in the Supplementary materials (Table S2).

Since EtOH, which is the most commonly used alcohol in drug formulations because of its low toxicity, produced the smallest PF14 particles among the used solvents, we selected the respective stock solution for further refinement, i.e., for supplementation with DMSO. DMSO is a polar aprotic organic solvent that successfully dissolves various compounds, including hydrophobic substances that are insoluble in aqueous solutions. DMSO can legally be used in therapeutic formulations up to a 10% (*v*:*v*) concentration, and its side effects are considered not significant. According to the FDA, both EtOH and DMSO are class III solvents, i.e., considered the safest with minimal toxicity. Moreover, DMSO is also known to facilitate the cellular internalisation of many compounds, including macromolecules, such as CPPs [61] and their constructs with cargo [62]. The dissolution of PF14 in the EtOH/DMSO (9/1, *v*/*v*) mixture and further 10-fold dilution in water led to the formation of even slightly smaller peptide nanoparticles than with the pure ethanol, i.e., with about a 100 nm diameter (Figure 1A). This suggests that DMSO may contribute to the dissolution of PF14 in EtOH by weakening the hydrophobic peptide–peptide interactions, e.g., the interactions between the stearyl groups. In addition, after the dilution of the PF14 stock solution in water, DMSO could also contribute to the microenvironment around/inside the peptide particles [36], whose formation is triggered by condensation in an aqueous milieu that leads to phase separation [63].

The phase separation induced by the transfer of PF14 from an organic solvent phase into an aqueous environment is less efficient than in the case of peptides with fluoroalkane modification [64]. In order to induce a more pronounced phase separation, we analysed the effect of the inclusion of excipients into the PF14 particles. Organic carbonates that show excellent protein solvation and stabilisation properties [65] and have limited solubility in aqueous solutions were, therefore, analysed for the ability to facilitate the assembly of PF14 nanoparticles and increase their homogeneity. Among the analysed organic carbonates, the cyclic compounds contributed to the homogeneity of PF14 particles. Trimethylene carbonate had the strongest stabilising effect on the peptide nanoparticles, followed by ethylene carbonate, whereas glycerol-1,3-carbonate did not contribute to the formation of particles (data not shown).

Trimethylene carbonate (TMC) is a cyclic ester with a six-member ring structure, which is most often used for the preparation of biodegradable polymers used for biomedical applications [66]. The polymerisation of TMC results in the formation of poly(trimethylene carbonate) (PTMC), a highly promising component for soft-tissue engineering and drug delivery [67]. However, here, to assist the formation of PF14 nanoparticles in water, we added a very low amount of TMC (below 1%) to the mixture of EtOH and DMSO, which was used to dissolve the PF14 peptide. We varied the concentration of TMC in the peptide stock solution from 0.1% to 1% and analysed the hydrodynamic diameter of the resulting PF14 particles (Figure 1B). The addition of 1% TMC to the PF14 solution produced reasonably low hydrodynamic diameters and high homogeneity in the water particles (PDI around 0.2). Lowering the TMC concentration, surprisingly, increased the diameter of the PF14 particles and strongly increased their heterogeneity, especially at the 0.5–0.6% concentration. The homogeneity and suitable size of the particles were regained at a 0.4% concentration of TMC, which we considered optimal for PF14 because the NA cargo should not be too tightly condensed into nanoparticles with the peptide, which could happen at a 1% TMC concentration. Thus, after translocation into the cells, oligonucleotides would more probably dissociate from not-overly-stable PF14 nanoparticles in order to be active. Therefore, PF14 dissolved in EtOH90/DMSO10/TMC0.4 was used to analyse the properties of the particles formed by the peptide upon complexation with SCO and siRNA (Table S3). The advantageous properties of PF14 nanoparticles were not compromised by complexation with SCO or siRNA or by the further supplementation of particles with divalent metal ions, i.e., Ca^2+^ and Mg^2+^ (Table S3). The metal ions have earlier been shown to increase the efficiency of PF14-NA complexes multiple fold and were, therefore, included in this study [51]. Particles of PF14 in complex with SCO were of similar size to the particles that consisted of the peptide only (112 and 119 nm, respectively). Surprisingly, the particles prepared using PF14 and siRNA had lower hydrodynamic diameters (87 nm) than the peptide particles, suggesting the reorganisation of the latter upon complex formation with oligonucleotides. The addition of CaCl_2_ and MgCl_2_ to the CPP–NA complexes did not significantly alter their size, except for the PF14-siRNA-Mg^2+^ particles, which were substantially larger than the corresponding particles without MgCl_2_. All of the nanoparticles had a positive zeta potential (Table S3), with SCO-containing complexes possessing higher charge than the siRNA-containing ones.

### 3.2. Dissolution of PF14 in the Mixture of Polar Organic Solvents Does Not Compromise the Ability of Peptide to Transduce Oligonucleotides into Cells

DLS analyses indicated that PF14 dissolved in various organic solvents led to the formation of smaller and more homogeneous particles in an aqueous milieu as compared to the peptide dissolved directly in water. In order to test whether the nanoparticles formed by NAs and PF14 dissolved in organics can mediate the efficient delivery of the cargo molecules and induce biological effects in cells, we first harnessed a model system based on splicing switching. The delivery of splicing switching oligonucleotide SCO-705 into HeLa pLuc 705 reporter cells abolishes the aberrant splicing of pre-mRNA and thereby rescues the expression of luciferase, whose activity correlates very well with the concentration of SCO in cell cytosol [52]. First, we prepared PF14 solutions in alcohol–DMSO (9/1) mixtures and formed the respective nanoparticles with SCO. Surprisingly, all of the PF14 solutions prepared in the alcohol–DMSO mixture yielded a slightly higher efficiency for splicing correction in HeLa pLuc 705 cells than the particles prepared with water-dissolved peptides when the particles were complemented with Ca^2+^ or Mg^2+^ ions (Figure S1). On the contrary, the particles containing only PF14 and SCO showed rather similar effects on splicing switching in the case of all of the different peptide stock solutions used. As all of the alcohol/DMSO mixtures resulted in similar activity of SCO-PF14 nanoparticles, we kept using EtOH as the most biocompatible one.

**Figure 2:**
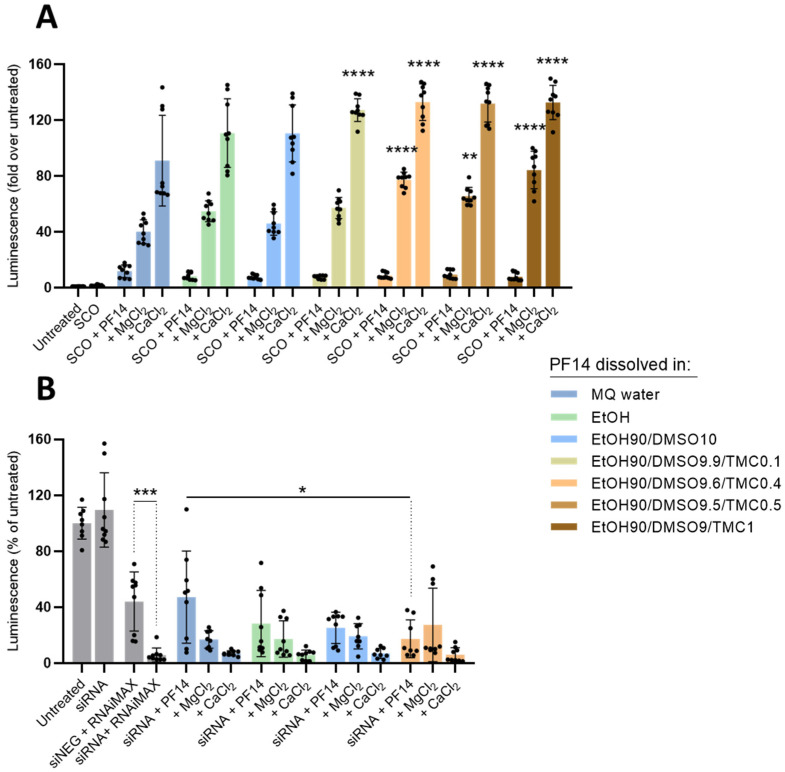
Effect of nanoparticles prepared from SCO or siRNA and differently dissolved PF14 on splicing correction and luciferase silencing, respectively. The 1 mM stock solutions of PF14 were prepared by dissolving the peptide in different solvents and their mixtures, as indicated in legends (proportions are given as *v*:*v*). (**A**) HeLa pLuc 705 cells were incubated for 24 h with solutions containing SCO alone (100 nM), nanoparticles of SCO and PF14 taken at MR 5 (PF14:SCO) with or without the addition of 3 mM CaCl_2_ or MgCl_2_. (**B**) U87 MG-Luc2 cells were incubated for 48 h with solutions containing siRNA alone (15 nM), nanoparticles of siRNA and PF14 taken at MR 34 (PF14:siRNA) with or without the addition of 3 mM CaCl_2_ or MgCl_2_. As a positive control, siRNA was transfected with Lipofectamine RNAiMAX. In all cases, as a negative control, the cells were incubated with a medium containing 10% (*v*:*v*) of MQ water (“Untreated”). siNEG—negative, i.e., non-targeting siRNA. Each dataset represents mean ± SD of technical replicates (shown as black dots) from three independent experiments. Data were analysed using one-way ANOVA with post-hoc Tukey’s test. Asterisks indicate statistically significant difference compared to the same solution from “MQ water” group (**A**) or between indicated datasets (**B**), * *p*-value < 0.05, ** *p*-value < 0.005, *** *p*-value < 0.0005, **** *p*-value < 0.0001.

Next, in the same way, we analysed the PF14 stock solutions prepared in MQ, EtOH, EtOH90/DMSO10, and EtOH90/DMSO/TMC, with varying percentages of TMC (Figure 2). As can be seen in Figure 2A, all of the SCO-PF14 nanoparticles prepared with PF14 dissolved in organic solvents showed activity similar to the ones prepared using water stock. The inclusion of biocompatible divalent cations, Ca^2+^ and Mg^2+^, into the nanoparticles strongly increased the SCO delivery efficiency of PF14, as we observed earlier [51]. Although the increase was observed in the case of all the PF14 stocks used, it was more prominent and consistent in the case of the solutions that contained TMC, and respective SCO-PF14-Ca^2+^/Mg^2+^ particles led to a significantly higher splicing correction than the ones prepared with water-dissolved PF14. Although both DLS (Figure 1) and splicing correction assay (Figure 2A) showed that PF14 stocks with 0.4% and 1% of TMC provided equally small and homogeneous particles, the high activity of the cargo and good reproducibility of the results, we selected PF14 dissolved in an EtOH90/DMSO9.6/TMC0.4 mixture to be used in further experiments.

Next, we assessed whether PF14 dissolved in the polar organic solvent mixtures could perform at the same level as the water-dissolved peptides in the delivery of another NA oligomer with different biological effects, siRNA. Silencing RNA introduced into luciferase- overexpressing U87 MG-Luc2 cells with PF14 strongly suppressed luciferase expression, even at a 15 nM siRNA concentration (Figure 2B). The nanoparticles formed by siRNA and PF14 dissolved in the EtOH–DMSO mixture were slightly more efficient than those assembled from peptide stocks made in water or EtOH, reducing target protein expression by about 70%. Importantly, Ca^2+^ and mostly also Mg^2+^ ions increased the luciferase-silencing effect of PF14-siRNA nanoparticles for all of the differently dissolved PF14 samples. Importantly, none of the tested solutions significantly affected the viability of HeLa pLuc 705 or U87 MG-Luc2 cells (Figure S2).

Based on these results, we can conclude that the dissolution of PF14 in the mixture of the selected polar organic solvents does not compromise the cellular delivery of oligonucleotides, sometimes providing even slightly higher biological effects, and the efficiency of the formed nanoparticles can be substantially increased by the inclusion of divalent metal ions into the nanoparticles.

### 3.3. Electron Microscopy Confirms the Beneficial Effect of Dissolving PF14 in Organic Solvents, Compared to Dissolving in Water

**Figure 3:**
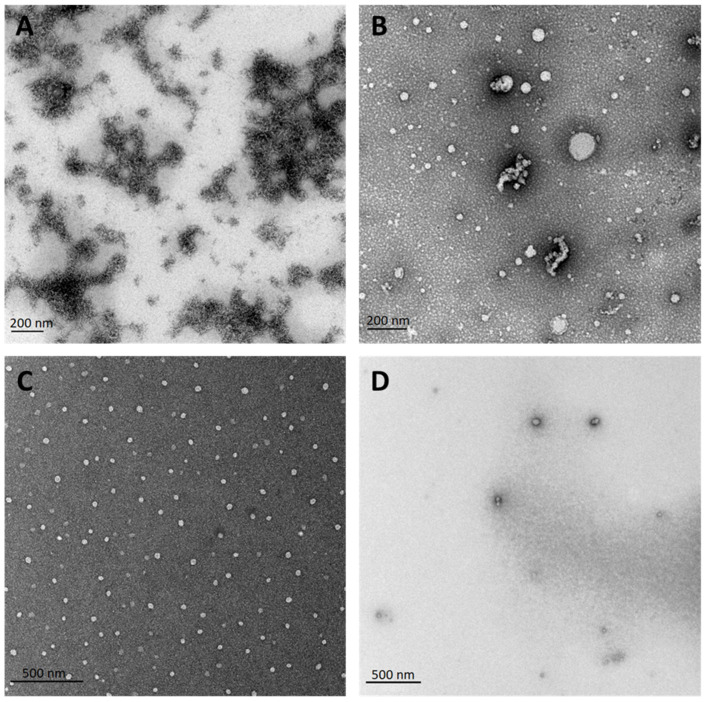
Morphology of particles formed by differently dissolved PF14 as analysed by TEM. The 1 mM stock solutions of PF14 were prepared by dissolving the peptide in different solvents and their mixtures: MQ water (**A**), EtOH (**B**), EtOH90/DMSO10 (**C**) or in EtOH90/DMSO9.6/TMC0.4 (**D**), and resulting particles were analysed by TEM. All proportions are given as *v*:*v*.

Now, when both the DLS (Figure 1) and activity data (Figure 2 and Figure S1) showed that PF14 dissolved in organic solvents yields particles with advantageous physical properties and delivers NAs into cells with an efficiency comparable with or even superior to water-dissolved PF14 we analysed the TEM particles formed in the water milieu using the following PF14 stocks: MQ, EtOH, EtOH90/DMSO10, and EtOH90/DMSO9.6/TMC0.4 (Figure 3). In good concordance with the DLS analysis data, PF14 dissolved in water was mostly detected in electron microphotos as large aggregates or conglomerates of very large elongated micelles up to 500 nm or even larger sizes (Figure 3A). PF14 dissolved in EtOH assembled into more regular particles upon dilution in water, i.e., under the conditions used to assemble PF14 nanoparticles with oligonucleotide cargo (Figure 3B). The majority of the peptide was organised in spherical nanoparticles of about 30 to 100 nm diameters, and some smaller particles assembled into grape or necklace-like clusters, whereas very large aggregates were not detected in the TEM images.

The solvent mixture of EtOH and DMSO (9/1) seemed to be almost optimal for the dissolution of PF14 since rather homogeneous nanoparticles with <100 nm diameter formed upon the dilution in water, and no aggregates or clusters of nanoparticles were detected in microphotos (Figure 3C). The particles formed by PF14 dissolved in EtOH90/DMSO9.6/TMC0.4 were of similar size and morphology compared to the EtOH90/DMSO10 stock (Figure 3D). The numerical values of all particles’ diameters calculated from electron micrographs of Figure 3 can be found in the Supplementary Materials (see Table S4).

### 3.4. Confocal Microscopy Analysis Corroborates Efficient Oligonucleotide Delivery and Splicing Correction with PF14 Dissolved in the Mixture of Organic Solvents

In order to visualise the transfection and intracellular distribution of SCO, as well as the splicing correction triggered by it, we performed confocal microscopy of HeLa EGFP 654 cells. In this cell line, analogous to HeLa pLuc 705, the mutated intron of beta-globin is included in the genome, interfering with the expression of EGFP protein. The pre-mRNA of EGFP contains an aberrant splicing that induces mutation in position 654, and added SCO-654 blocks the mutated site, restoring normal splicing and rescuing EGFP expression.

**Figure 4:**
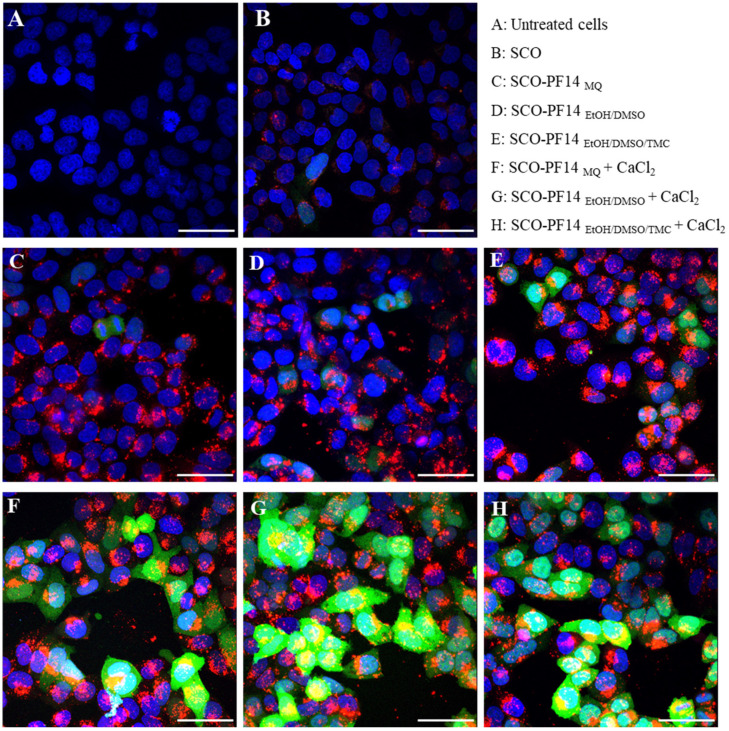
Internalisation and biological effect of nanoparticles containing SCO, differently dissolved PF14 and CaCl_2_. HeLa EGFP 654 reporter cells were incubated with nanoparticles of 180 nM SCO-654, 20 nM Cy5-SCO-654, 1 μM PF14 and 3 mM CaCl_2_ for 24 h. Cells were fixed, and specimens were analysed with Olympus FluoView FV1000 confocal microscope. Cells were either left untreated (**A**), incubated with the two SCOs (**B**), the SCO-PF14 nanoparticles (**C**–**E**) or the SCO-PF14-Ca^2+^ nanoparticles (**F**–**H**). The following PF14 stocks were used: PF14 dissolved in MQ water (**C**,**F**); PF14 dissolved in EtOH90/DMSO10 mixture (**D**,**G**); and PF14 dissolved in EtOH90/DMSO9.6/TMC0.4 mixture (**E**,**H**). Rescued EGFP expression is shown in green. Cell nuclei were visualised with DAPI (blue). For tracking cellular association and internalisation of oligonucleotide, Cy5-labelled SCO was used (red). The merged images of all confocal layers are presented. Scale bar: 50 µm.

In addition to the particles of 1 µM PF14 and 200 nM SCO (Figure 4C–E), particles with the addition of 3 mM CaCl_2_ were included (Figure 4F–H). As expected, all of the PF14 stocks tested induced equally efficient transduction of SCO-Cy5 (red) into cells, independent of the addition of CaCl_2_. However, a substantial increase in EGFP expression induced by SCO-654 could be observed when CaCl_2_ was added to the particles. In the microscopy images, no significant changes in the transfection or expression efficiency or in the intracellular distribution of SCO could be detected when different PF14 stock solutions were used. As expected, the morphology of the cells was not affected by any of the solutions used (Figure S3).

For better quantification of the induced splicing correction in HeLa EGFP 654 cells, we next applied flow cytometry analysis. The incubation of HeLa EGFP 654 cells with nanoparticles analogous to the ones used in the confocal microscopy experiment (Figure S4) revealed significant advantages of CaCl_2_/MgCl_2_-supplemented SCO-PF14 nanoparticles prepared with PF14 dissolved in organic solvent mixtures compared to water-dissolved PF14 (Figure S4A). In the case of PF14 dissolved in MQ water, nanoparticles supplemented with the salts provided an average 4.34× increase in EGFP expression compared to the untreated cells. However, in the case of PF14 dissolved in EtOH90/DMSO and EtOH90/DMSO9.6/TMC0.4, salt-supplemented nanoparticles provided 6× and 6.93× increase in fluorescence, respectively, being significantly more efficient than their counterparts prepared with water-dissolved PF14. It is worth mentioning that, unlike PF14 complexes with siRNA and SCO-705, PF14-SCO-654 nanoparticles benefited equally from the addition of CaCl_2_ and MgCl_2_ (Figure S4A). As expected, there were no remarkable differences in Cy5 fluorescence intensity between the differently dissolved PF14 solutions, suggesting that the internalisation of SCO-delivering nanoparticles was not substantially influenced. The only exceptions were MgCl_2_-supplemented PF14-SCO particles prepared with PF14 dissolved in EtOH, which yielded slightly lower transfection than other peptide specimens (Figure S4B).

Altogether, these results indicate that PF14 dissolved in organic solvents, especially in their mixtures, can be superior to water-dissolved PF14 in a fluorescence rescue assay based on splicing correction/switching.

### 3.5. Other Organic Solvents Can Also Be Beneficial for Dissolving PF14, and Hydrophobic CPPs

In addition to the typically used alcohols, such as MeOH, EtOH, iPrOH, butanol, and DMSO, we also tested whether other commonly used organic polar aprotic solvents, such as DMF and THF alone or in combination with different alcohols could be used to dissolve PF14 and prepare biologically highly active nanoparticles with SCO (Figure S5). In general, SCO-PF14 nanoparticles supplemented with Ca^2+^ showed higher splice-correcting effects if DMF and THF were used for the preparation of peptide stock solutions compared to the particles formed using peptides dissolved in water. DMF- and THF-containing PF14 solutions had transfection efficiency comparable to PF14 dissolved in EtOH90/DMSO9.6/TMC0.4, enabling up to a 130× increase in reporter protein activity/luminescence when supplemented with CaCl_2_. Surprisingly, a stock prepared in MeOH90/DMF10 almost completely lacked activity, suggesting that this particular solvent system is not suitable for dissolving PF14 for biological assays. Other DMF- and THF-containing PF14 stock solutions had similar activity, with pure DMF and THF10/EtOH90 being superior to other combinations. However, it should be mentioned that the FDA classifies both DMF and THF as class II solvents and, therefore, the harnessing of colloidal nanoparticles that contain these solvents is limited, especially when considering the high efficiency of nanoparticles formed with EtOH solutions of PF14. Correspondingly, the presence of DMF and THF in the respective solutions of PF14-SCO nanoparticles interfered to some extent with the viability of both HeLa pLuc 705 and U87 MG-Luc2 cells (Figure S6), with 100% THF used as a solvent being the most cytotoxic for both cell lines and 100% DMF also showing higher-than-average toxicity in HeLa pLuc 705.

After achieving remarkable changes in the activity of PF14 in different co-solvent systems in SCO transfection, we were interested in assessing whether this strategy of dissolving is also applicable to other peptides. For this, we examined two other well-known hydrophobic and fatty-acid-modified CPPs, which have been shown to self-assemble into nanoparticles with oligonucleotide cargo analogously to PFs, and could, in principle, aggregate in aqueous solutions. We included in our experiments hPep3 peptide, which showed promising results during SCO transfection due to its increased hydrophobicity [68], and CADY, which is a known efficient siRNA transporter [69,70,71]. We prepared nanoparticles of hPep3 and SCO in an analogous way to PF14-SCO nanoparticles, also using MR 5 (hPep3:SCO, 100 nM SCO) and adding 3 mM CaCl_2_ or MgCl_2_. All three hPep3 stocks dissolved in organic solvents showed SCO transfection efficiency and reporter-protein expression in cells at a similar level with water-dissolved peptides (Figure S7A). However, the reporter-protein expression achieved with the hPep3–SCO complexes remained lower compared to PF14-mediated delivery. Nanoparticles of CADY with siRNA were prepared at MR34 and complemented with 3 mM Ca^2+^/Mg^2+^, as mentioned previously, for PF14 complexes. All of the solutions of CADY, both prepared in water and organics, showed similar siRNA transfection and target protein knock-down (Figure S7B). No substantial cytotoxicity was detected in the case of hPep3 and CADY nanoparticles (Figure S8). We did not include in our experiments cationic CPPs that dissolve very well in water and are not prone to self-aggregate since we considered that these do not benefit from dissolution in an organic solvent.

### 3.6. Nanoparticles of PF14 and Its Complexes with SCO Are Spherical

After optimising the dissolution of PF14 regarding the activity of nanoparticles prepared with SCO and analysing their hydrodynamic diameter, homogeneity, and zeta potential using DLS, we continued with more detailed analyses of the properties of such particles. First, we assessed whether the changes in the secondary structure of PF14 were introduced upon association with SCO. Using circular dichroism spectroscopy (CD), we confirmed that in water, PF14 folds into an α-helical structure notwithstanding the solvent used for the initial dissolution of the peptide (Figure S9). There are, however, clear differences between the CD spectra for the different particles. The spectra for purely α-helical peptides cross the zero line around 203 nm, such as the spectrum of PF14 in complex with SCOs and 3 mM CaCl_2_ (Figure S10C). The spectra that cross the zero line below 203 nm, as most of the spectra seen in Figures S9 and S10 do, reflect peptides with a combination of α-helical and random-coil secondary structures. This is particularly clear for PF14 dissolved in MQ water only (Figure S9A), where the spectrum crosses the zero line below the 200 nm mark. Furthermore, typical α-helical CD spectra display a strong minimum at 208 nm and a weaker minimum at 222 nm. The [θ222]/[θ208] ratio is around 0.7 for isolated α-helical structures, while higher values for the [θ222]/[θ208] ratio—up to around 1:1 ratio—indicate that coil–coil interactions (also known as helix supercoiling) have formed [72]. The unusual spectrum shown in Figure S10D likely reflects a combination of α-helical and β-sheet structures. It should be noted that the overall intensities are different for the different samples, even though the peptide concentrations are the same. The lower signal intensities observed in, e.g., Figure S9A,C are most likely caused by the formation of large aggregates that either precipitate out of the solution or are too large to be penetrated by the CD light beam, in both cases, effectively reducing the observable peptide concentration. Surprisingly, upon interaction with SCO, the structure of PF14 was not reorganised, and it retained an α-helical structure, as characterised by CD spectroscopy (Figure S10). We believe that upon association with SCO (as well as other types of bioactive NA molecules), PF14 nanoparticles may partly dissociate to form nanocomplexes using electrostatic and hydrophobic interactions, whereas the secondary structure of the peptide is not reorganised.

**Figure 5:**
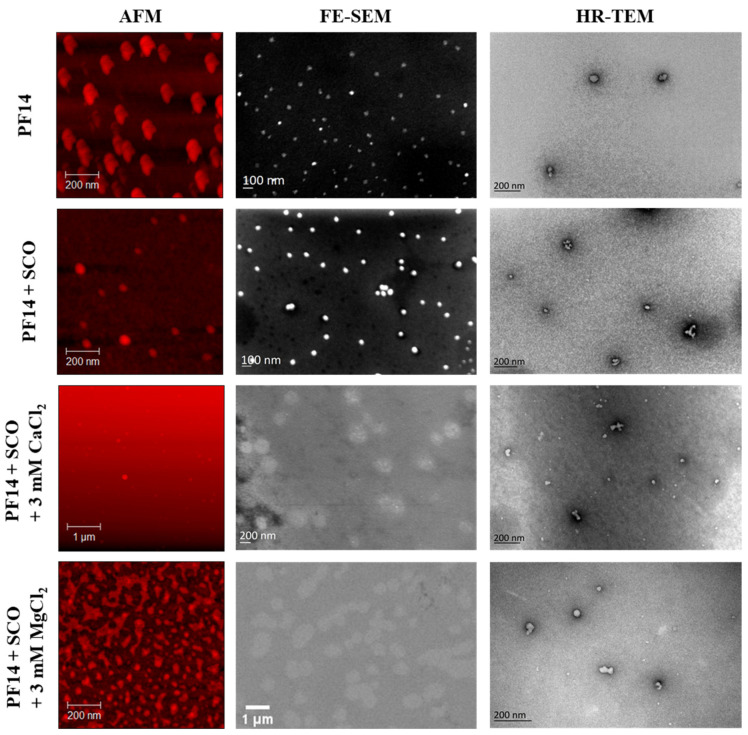
Morphology of particles formed by PF14 dissolved in EtOH90/DMSO9.6/TMC0.4 and its complexes with SCO and divalent metal ions added in the form of CaCl_2_ and MgCl_2_. Nanoparticles were prepared by mixing SCO and PF14 at MR 10 and adding CaCl_2_ and MgCl_2_ after 15 min. After a total of 30 min of incubation, solutions were analysed by AFM, field emission SEM (FE-SEM), or high-resolution TEM (HR-TEM).

Earlier, we showed by DLS and TEM analyses that PF14 dissolved in a solvent mixture of optimal composition (EtOH90/DMSO9.6/TMC0.4) formed nanoparticles upon dilution into an aqueous solution (Figure 1 and Figure 3). However, negative staining of specimens with aqueous uranyl acetate of low pH could influence the morphology of the detected particles [73], and the particles detected by DLS in solution may not be stable [74]. Therefore, to analyse whether the morphology of the self-assembled nanostructures formed upon the complexation of SCO with PF14 is favourable for the uptake by cells, we harnessed a battery of various microscopic techniques. We applied atomic force microscopy, scanning electron microscopy, and transmission electron microscopy (Figure 5) for a detailed analysis of nanoparticles’ morphology since these are commonly used to characterise nanoparticles in parallel with DLS. All, AFM, SEM, and TEM analyses confirmed that the PF14-SCO nanoparticles were spherical and with a diameter in the range of 40–60 nm (Figure 5, Table S5). The high homogeneity and small size of PF14-SCO nanoparticles detected by these biophysical methods are in very good concordance with their ability to trigger biological responses in living cells (Figure 2).

The inclusion of biologically relevant divalent cations Ca^2+^ and Mg^2+^ into PF14 nanoparticles with oligonucleotides strongly increased the biological effect of the latter inside the cells (Figure 2), consistent with earlier reports [48,51,75,76,77,78,79,80,81]. Therefore, we next analysed the morphology of metal ion-complemented PF14-SCO nanoparticles by AFM, SEM, and TEM (Figure 5, Table S5). After the inclusion of Ca^2+^ ions, the size of PF14-SCO nanoparticles increased to about 100–200 nm, and they retained a rather spherical shape, as seen in the AFM and SEM images (Figure 5). In the specimens stained with uranyl acetate (UA), TEM visualised the clusters of particles of similar size and also small nanoparticles (Figure 5). We consider it plausible that in the acidic milieu of uranyl acetate (pH around 4.5), used to stain the specimen, the PF14-SCO-Ca^2+^ nanoparticles could partly dissociate. The small nanoparticles detected in the UA-stained PF14-SCO-Ca^2+^ specimens have a similar size to the PF14-SCO particles formed without Ca^2+^/Mg^2+^, as seen in the TEM microphotos. This suggests that calcium ions are less strongly associated with PF14-SCO-Ca^2+^ nanoparticles compared to the interaction of PF14 with SCO. Therefore, in acidic conditions, calcium ions can leave the PF14-SCO-Ca^2+^ particle, leading to the (re)formation of PF14-SCO particles. Our suggestion is supported by a similar behaviour of PF14-SCO-Mg^2+^ nanoparticles. Their size is about 100 nm, as measured by DLS [51] (Table S3), but increases to several hundreds of nanometres when analysed by AFM and SEM at a neutral pH (Figure 5). However, in an acidic milieu, the size of PF14-SCO-Mg^2+^ nanoparticles decreases to about 50 nm, which is analogous to Ca^2+^ ions-containing particles (Figure 5). We assume that a partial protonation of SCO in an acidic milieu reduces its negative charge and could explain the facilitated dissociation of Ca^2+^/Mg^2+^ ions from PF14-SCO-Ca^2+^/Mg^2+^ particles and their further disintegration to smaller PF14-SCO nanoparticles. The biological effect of Mg ions-containing PF14-SCO nanoparticles was lower than that of the Ca-supplemented form. Expectedly, PF14-SCO-Mg nanoparticles were less well defined and differed in morphology, as revealed by AFM and SEM analyses (Figure 5, Table S5), corroborating results published earlier [51].

In order to analyse whether supplemented Ca^2+^ and Mg^2+^ ions are indeed associated into PF14-SCO nanoparticles, we used energy-dispersive X-ray spectroscopy (EDX) (Figure S11 and Table S6). EDX analysis confirmed the presence of Ca^2+^ and Mg^2+^ ions in nanoparticles assembled of PF14, SCO and CaCl_2_ or MgCl_2_, respectively.

We may conclude that when PF14 was dissolved in an optimal solvent mixture, it assembled into spherical nanoparticles after dilution in an aqueous media, and the aggregation of the peptide was not detected by the used battery of biophysical analysis methods. The spherical morphology of PF14 particles was retained after complex formation with the oligonucleotide cargo, and even after supplementation of PF14-SCO nanoparticles with Ca^2+^ or Mg^2+^ ions that strongly increase the bioavailability of cargo inside living cells.

## 4. Conclusions

In the current study, we have elaborated a new approach for formulating nonaggregating and uniformly sized CPP–oligonucleotide nanoparticles. Our results showed that the cosolvent approach used to dissolve hydrophobic CPP in organic solvents of high biocompatibility enabled better control of the size and aggregation of the nanoparticles compared with dissolving the peptide in water. Alcohols, such as EtOH or iPrOH, form hydrogen bonds with water and help to weaken the interactions between the peptide and water, modulating the morphology of peptide-formed particles [82,83], yielding smaller and more uniform particles than the ones formed by water-dissolved peptides. In addition to alcohols, we added DMSO to the peptide stock solutions, which is a highly potent solvent for dissolving hydrophobic compounds. As shown earlier, DMSO, in addition to increasing solubility, can also facilitate cell permeability of nanoparticles by forming hydrophilic pores in the lipid bilayer [84,85], thus isolating the nanoparticles from the hydrophobic environment of the plasma membrane [86]. Finally, we added a small amount of biodegradable organic carbonate, TMC [66], to the aforementioned mixtures in order to induce more efficient phase separation of CPP nanoparticles or their complexes with oligonucleotide from an aqueous milieu. We found that TMC, in combination with alcohol and DMSO, yielded peptide nanoparticles of highly suitable size and increased the homogeneity of the nanoparticle population, and enhanced the biological effect of CPP-NA particles in living cells.

The solvent mixture that we optimised here to dissolve PF14 enabled us to assemble highly uniform nanoparticles of peptides and oligonucleotides, which provided comparable or, in some cases, increased expression of the reporter protein under cell culture conditions, compared to water, a solvent commonly used for dissolving peptides. Furthermore, an important advantage of this solvation method is that it allows the long-term storage of the peptide stock solutions without changes/loss of its efficiency/activity. We observed more consistent results while using non-freezing stocks prepared in organic solvent mixtures compared to water stocks that have to be either stored at +4 °C or thawed upon each use, with both of these approaches facilitating aggregation and changes in the properties of the peptide.

The biological effect of oligonucleotide cargo was further increased by the inclusion of Ca^2+^ and Mg^2+^ ions into the aforementioned CPP–oligonucleotide nanoparticles. Importantly, the complementation of nanoparticles with CaCl_2_ only marginally increased the size of the particles, making these highly biocompatible for in vitro and possibly for in vivo applications. Added calcium ions seem to dissociate from the complemented nanoparticles in an acidic milieu, which could even be relevant to the escape of CPP–ON nanoparticles from entrapment in acidic endosomes. The applicability of the strategy proposed here for the cellular delivery of NA molecules with higher molecular weight, such as mRNA and pDNA, is not clear yet and is the focus of our future studies.

## Data Availability

All data supporting the findings of this study are available within the article and its supplemental information or from the corresponding author upon reasonable request.

## Supplementary Materials

The following supporting information can be downloaded at: https://www.mdpi.com/article/10.3390/pharmaceutics15020396/s1, Table S1: CPPs used in the study, their sequences, charge and number of amino acid residues; Table S2: Characterisation of particles forming from PF14 dissolved in different solvents by DLS; Table S3: Hydrodynamic diameter, polydispersity index and zeta potential of nanoparticles forming upon complexation of PF14 with SCO (at MR 5) or siRNA (at MR 34) with addition of Ca^2+^ or Mg^2+^; Table S4: Average diameter of nanoparticles formed by differently dissolved PF14, which was calculated from HR-TEM images using the ImageJ software; Table S5: Average diameter of nanoparticles prepared of 100 nM SCO, 1 µM PF14 and CaCl_2_ or MgCl_2_, which was calculated from AFM, FE-SEM, and HR-TEM images using ImageJ software; Table S6: Numerical results of EDX analysis of nanoparticles assembled from PF14 in EtOH90/DMSO9.6/TMC0.4, SCO and CaCl_2_ or MgCl_2_; Figure S1: Effect of nanoparticles prepared from SCO-705 and differently dissolved PF14 on splicing correction; Figure S2: Viability of HeLa pLuc 705 (A) or U87 MG-Luc2 (B) cells after incubation with complexes of differently dissolved PF14 and SCO-705 (A) or siRNA (B); Figure S3: Bright field images of cells after incubation with nanoparticles containing SCO, differently dissolved PF14 and CaCl_2_; Figure S4: Transfection of and splicing correction with SCO-654 delivered with differently dissolved PF14; Figure S5: The effect of nanoparticles prepared of SCO-705, differently dissolved PF14 and Ca^2+^/Mg^2+^ ions on splicing correction; Figure S6: The effect of nanoparticles prepared of SCO (A) or siRNA (B), differently dissolved PF14 and Ca^2+^/Mg^2+^ ions on cell viability; Figure S7: Effect of cosolvent on tranfection of SCO and siRNA by hPep3 and CADY; Figure S8: Effect of nanoparticles prepared of SCO and hPep3 (A) or siRNA and CADY (B), on viability of HeLa pLuc 705 or U87 MG-Luc2 cells, respectively; Figure S9: Circular dichroism (CD) spectra reflecting the secondary structure of PF14 dissolved in MQ water (A), EtOH (B), EtOH90/DMSO10 (C), and in EtOH90/DMSO9.6/TMC0.4 (D); Figure S10: Circular dichroism (CD) spectra reflecting the secondary structure of PF14 (A), complex of PF14 with SCO (B); complex of PF14 with SCO and 3 mM CaCl_2_ (C); complex of PF14 with SCO and 3 mM MgCl_2_ (D) dissolved in EtOH; Figure S11: EDX spectra confirming the presence of Ca^2+^ (A) and Mg^2+^ (B) ions in nanoparticles assembled from PF14, SCO and CaCl_2_ or MgCl_2_, respectively.
