# Supplementary material for: Choosing an Optimal Solvent Is Crucial for Obtaining Cell-Penetrating Peptide Nanoparticles with Desired Properties and High Activity in Nucleic Acid Delivery
Source: Pharmaceutics. 2023 Jan 24;15(2):396. doi: 10.3390/pharmaceutics15020396 (PMC9963036; doi:10.3390/pharmaceutics15020396)
Supplement: Supplementary file 1 [file pharmaceutics-15-00396-s001.zip › pharmaceutics-2170421-supplementary.pdf]

pharmaceutics

Supplementary Materials: Choosing an Optimal Solvent is Crucial for Obtaining Cell-Penetrating Peptide Nanoparticles with Desired Properties and High Activity in Nucleic Acid Delivery

Abhijit Biswas, Maria Maloverjan, Kärt Padari, Aare Abroi, Margus Rätsep, Sebastian K. T. S. Wärmländer, Jüri Jarvet, Astrid Gräslund, Vambola Kisand, Rünno Lõhmus and Margus Pooga

Table S1. CPPs used in the study, their sequences, charge and number of amino acid residues. Ac = acetyl, (R8) = (R)-2-(7-octenyl)alanine, O – ornithine.

CPP	Sequence	Charge (pH 7.4)	Length (aa)
PF14	Stearyl-AGYLLGKLLOOLAAAALOOLL-amide	+5	21
CADY	Ac-GLWRALWRLLRSLWRLLWRA-cysteamide	+4	20
hPep3	H-L(R8)KLAKA(R8)AKLLKA(R8)AKAL-NH_2	+6	16

Table S2. Characterization of particles forming from PF14 dissolved in different solvents by DLS. Nanoparticles were prepared by diluting 1 mM PF14 stock solutions in different solvent mixtures in water 100× and incubating them for 30 min before measurement with Zetasizer Nano. Each number represents a mean (± SEM) of at least three independent measurements.

Solvent	Hydrodynamic diameter (nm)	Polydispersity index (PDI)
MQ water	319.0 ± 109.1	0.59
DMSO	279.2 ± 67.7	0.58
MeOH	326.5 ± 84.9	0.52
EtOH	102.8 ± 11.1	0.33
iPrOH	112.9 ± 18.5	0.30
EtOH90/DMSO10	87.9 ± 15.2	0.26
EtOH90/DMSO9/TMC1	131.2 ± 35.1	0.21
EtOH90/DMSO9.4/TMC0.6	274.7 ± 206.1	0.49
EtOH90/DMSO9.5/TMC0.5	269.5 ± 156.6	0.63
EtOH90/DMSO9.6/TMC0.4	118.9 ± 11.3	0.26
EtOH90/DMSO9.7/TMC0.3	341.7 ± 95.1	0.32
EtOH90/DMSO9.9/TMC0.1	327.2 ± 67.8	0.60

Table S3. Hydrodynamic diameter, polydispersity index and zeta potential of nanoparticles forming upon complexation of PF14 with SCO (at MR 5) or siRNA (at MR 34) with addition of Ca^{2+} or Mg^{2+}. Nanoparticles were prepared by 10× dilution of the PF14-SCO-Ca^{2+}/Mg^{2+} or PF14-siRNA-Ca^{2+}/Mg^{2+} complexes in water and analysed by DLS. Each number represents the mean value (± SEM) of at least three independent measurements. For all solutions, PF14 stock in EtOH90/DMSO9.6/TMC0.4 was used.

Sample	Hydrodynamic diameter (nm)	Polydispersity index (PDI)	Zeta potential (mV)
PF14 + SCO	112.3 ± 20.9	0.58	21.9 ± 2.3
PF14 + SCO + $CaCl_2$	133.2 ± 11.6	0.80	28.8 ± 1.7
PF14 + SCO + $MgCl_2$	91.1 ± 12.0	0.49	25.8 ± 1.2
PF14 + siRNA	86.6 ± 23.4	0.76	18.9 ± 3.0
PF14 + siRNA + $CaCl_2$	69.3 ± 23.7	0.67	10.6 ± 0.4
PF14 + siRNA+ $MgCl_2$	335.5 ± 63.9	0.70	17.6 ± 0.2

Table S4. Average diameter of nanoparticles formed by differently dissolved PF14, that was calculated from HR-TEM images (see Figure 3) using the ImageJ software.

Sample	Average diameter from HR-TEM (nm)
PF14 in EtOH	67 ± 39
PF14 in EtOH90/DMSO10	33 ± 4
PF14 in EtOH90/DMSO9.6/TMC0.4	49 ± 11

Table S5. Average diameter of nanoparticles prepared of 100 nM SCO, 1 μM PF14 and $CaCl_2$ or $MgCl_2$, that was calculated from AFM, FE-SEM and HR-TEM images (see Figure 5) using the ImageJ software. For all solutions, PF14 stock in EtOH90/DMSO9.6/TMC0.4 was used.

Sample	Avg. diameter from AFM (nm)	Avg. diameter from FE-SEM (nm)	Avg. diameter from HR-TEM (nm)
PF14	73 ± 5	36 ± 3	44 ± 12
PF14 + SCO	51 ± 13	57 ± 7	37 ± 7
PF14 + SCO + $CaCl_2$	122 ± 31	276 ± 23	31 ± 3
PF14 + SCO + $MgCl_2$	35 ± 8	608 ± 99	28 ± 4

Table S6. Numerical results of EDX analysis of nanoparticles assembled from PF14 in EtOH90/DMSO9.6/TMC0.4, SCO and $CaCl_2$ or $MgCl_2$.

Sample	Weight % of oxygen	Weight % of Ca	Weight % of Mg	Atomic % of oxygen	Atomic % of Ca	Atomic % of Mg
PF14 + SCO + $CaCl_2$	78.7 ± 7.6	21.3 ± 7.6	-	90.1 ± 4.0	9.9 ± 4.0	-
PF14 + SCO + $MgCl_2$	50.1 ± 9.4	-	30.6 ± 16.5	63.1 ± 8.6	-	25.2 ± 12.8

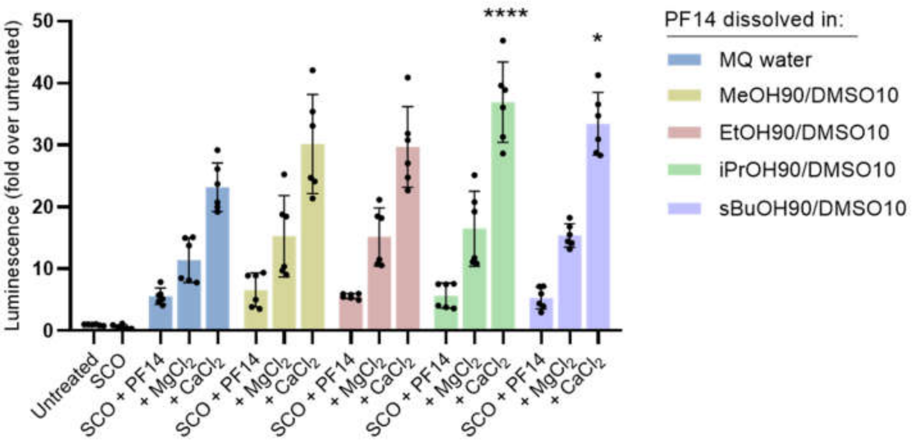

Figure S1. Effect of nanoparticles prepared from SCO-705 and differently dissolved PF14 on splicing correction. 1 mM stock solutions of PF14 were prepared by dissolving the peptide in different alcohols mixtures with DMSO (9/1, v/v). Nanoparticles of 1 μM SCO-705, 5 μM PF14 and 30 mM $MgCl_2$ or $CaCl_2$ were prepared by mixing the components in MQ water. After 30 min incubation, solutions were diluted 10-fold with cell culture medium and added to HeLa pLuc 705 cells. Luciferase activity was measured after 24 h of incubation. As a negative control, cells were incubated with a medium containing 10% (v:v) of MQ water („Untreated“). Data was analysed by one-way ANOVA with post-hoc Tukey's test. Asterisks indicate statistically significant difference, compared to the analogous solution from "MQ water" group, * p-value < 0.05, **** p-value < 0.0001.

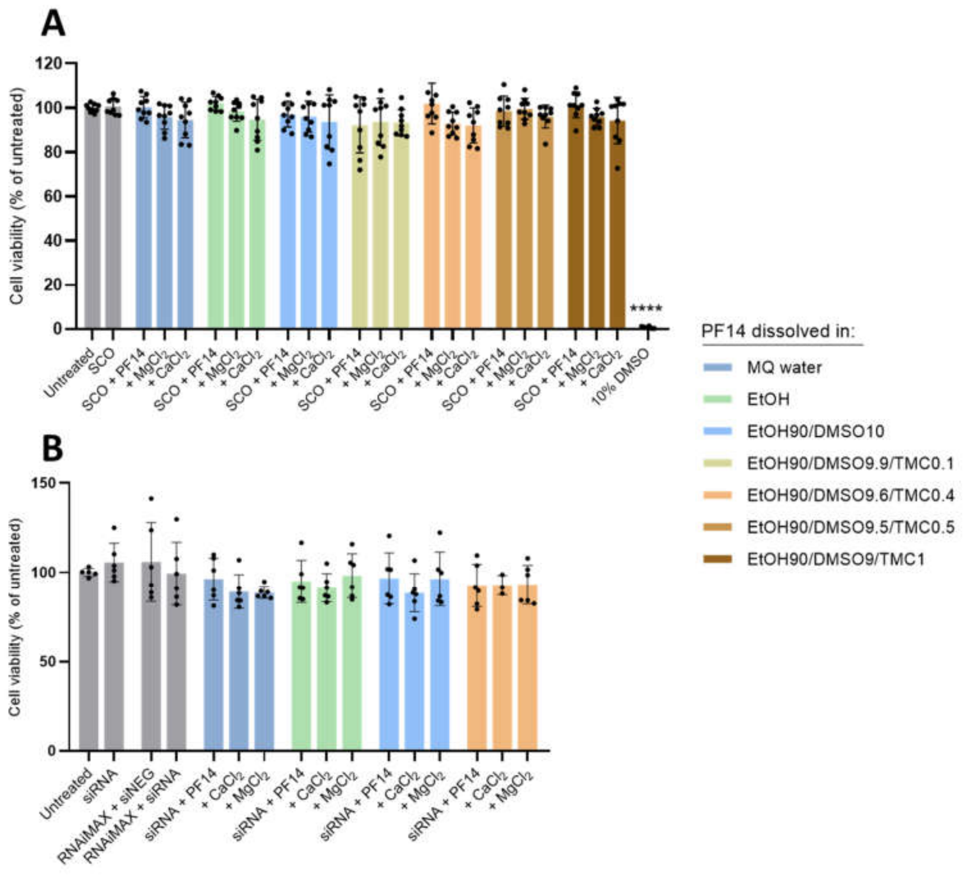

Figure S2. Viability of HeLa pLuc 705 (**A**) or U87 MG-Luc2 (**B**) cells after incubation with complexes of differently dissolved PF14 and SCO-705 (**A**) or siRNA (**B**). 1 mM stock solutions of PF14 were prepared by dissolving the peptide in different solvents and their mixtures, as indicated in the legend (proportions are given as v:v). (**A**) HeLa pLuc 705 cells were incubated for 24 h with solutions containing SCO alone (100 nM), nanoparticles of SCO and PF14 taken at MR 5 (PF14:SCO) with or without addition of 3 mM Ca^{2+} or Mg^{2+}. (**B**) U87 MG-Luc2 cells were incubated for 48 h with solutions containing siRNA alone (15 nM), nanoparticles of siRNA and PF14 taken at MR 34 (PF14:siRNA) with or without addition of 3 mM Ca^{2+} or Mg^{2+}. Viability of the cells was evaluated using WST-8 assay, and absorption of untreated cells was taken for 100%. Each dataset represents mean ± SD of technical replicates from three or two independent experiments, respectively. Data was analysed by one-way ANOVA with Dunnett's test.Asterisks indicate statistically significant difference, compared untreated cells, **** *p*-value < 0.0001.

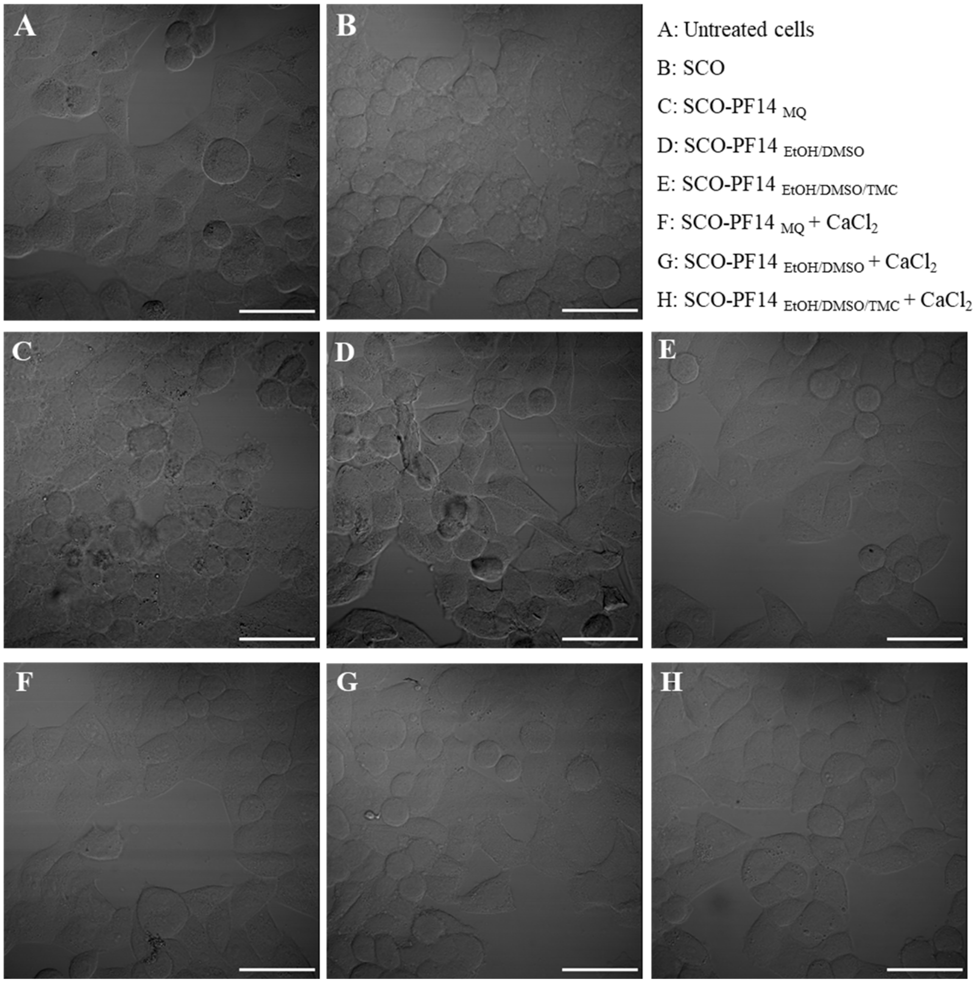

Figure S3. Bright field images of cells after incubation with nanoparticles containing SCO, differently dissolved PF14 and $CaCl_2$ (for fluorescent images, see Figure 4). HeLa EGFP 654 reporter cells were incubated with nanoparticles of 180 nM SCO-654, 20 nM Cy5-SCO-654, 1 μM PF14 and 3 mM $CaCl_2$ for 24 h. Cells were fixed and specimens were analysed with Olympus FluoView FV1000 confocal microscope. Cells were either left untreated (**A**), incubated with the two SCOs (**B**), the SCO-PF14 nanoparticles (**C**,**D**,**E**) or the SCO-PF14-Ca^{2+} nanoparticles (**F**,**G**,**H**). The following PF14 stocks were used: PF14 dissolved in MQ water (**C**, **F**); PF14 dissolved in EtOH90/DMSO10 mixture (**D**, **G**); and PF14 dissolved in EtOH90/DMSO9.6/TMC0.4 mixture (**E**, **H**). One confocal layer is presented. Scale bar 50 μm.

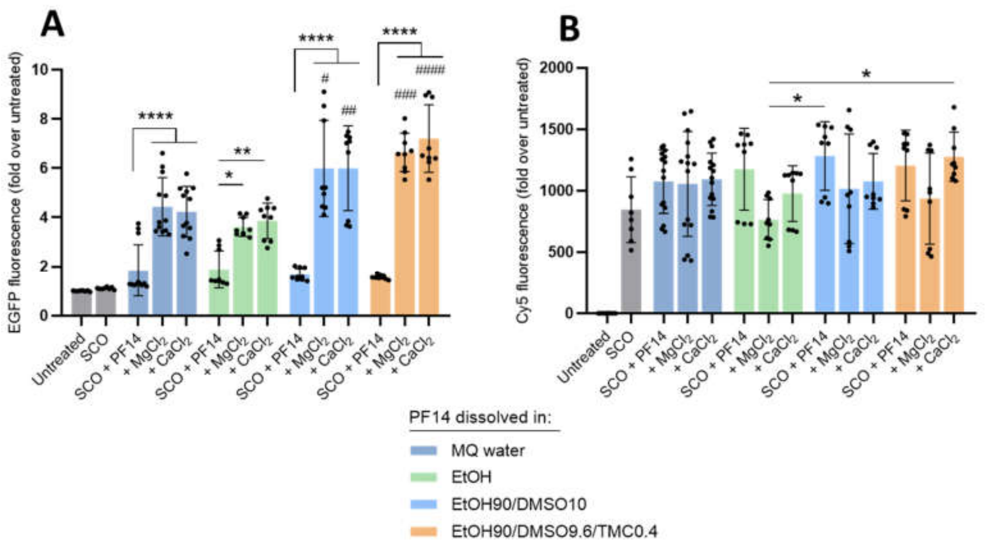

Figure S4. Transfection of and splicing correction in HeLa EGFP 654 cells with SCO-654 delivered with differently dissolved PF14. 1 mM stock solutions of PF14 were prepared by dissolving the peptide in MQ water and different organic solvents, as indicated in the legend (all ratios are given as v:v). HeLa EGFP 654 cells were seeded in 96-well plate and transfected with nanoparticles prepared of 90 nM SCO-654, 10 nM Cy5-SCO-654, 500 nM PF14 and 3 mM $CaCl_2$ or $MgCl_2$ next day. After 24 h of incubation, internalization of Cy5-labelled SCO (**B**) and splicing correction efficiency (**A**) were measured by Attune NxT flow cytometer. Each dataset represents mean ± SD of three independent experiments, with each dot corresponding to one technical replicate. The data was analysed by one-way ANOVA with post-hoc Tukey's test. Asterisks indicate statistically significant difference between two indicated datasets, * p-value < 0.05, ** p-value < 0.005, **** p-value < 0.0001. Hashes indicate statistically significant differences, compared to the analogous solution from "MQ water" group, # p-value < 0.05, ## p-value < 0.005, ### p-value < 0.0005, #### p-value < 0.0001.

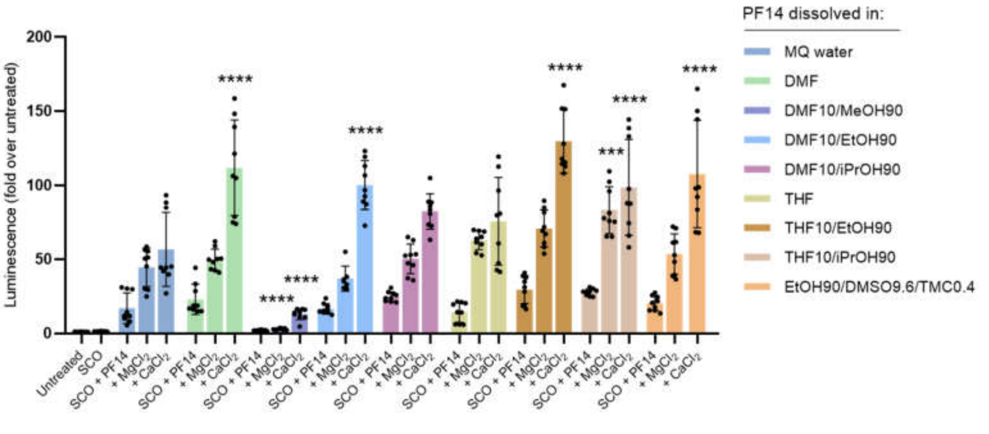

Figure S5. The effect of nanoparticles prepared of SCO-705, differently dissolved PF14 and Ca^{2+}/Mg^{2+} ions on splicing correction. 1 mM stock solutions of PF14 were prepared by dissolving the peptide in different solvents and their mixtures, as indicated in the legend (proportions are given as v:v). HeLa pLuc 705 cells were incubated for 24 h with solutions containing SCO alone (100 nM), nanoparticles of SCO and PF14 taken at MR 5 (PF14:SCO) with or without addition of 3 mM $CaCl_2$ or $MgCl_2$. Each dataset represents mean ± SD of technical replicates from three independent experiments. Data was analysed by one-way ANOVA with Tukey's test. Asterisks indicate statistically significant difference, compared to the analogous dataset from "MQ water" group, *** p-value < 0.0005, **** p-value < 0.0001.

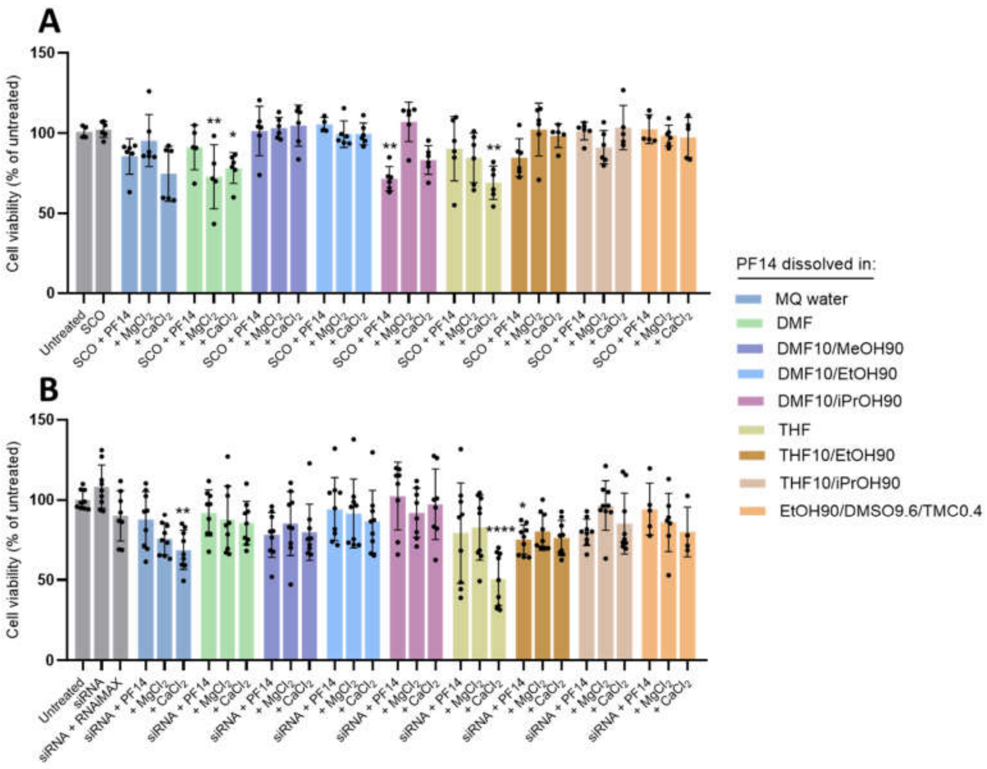

Figure S6. The effect of nanoparticles prepared of SCO (**A**) or siRNA (**B**), differently dissolved PF14 and Ca^{2+}/Mg^{2+} ions on cell viability. 1 mM stock solutions of PF14 were prepared by dissolving the peptide in different solvents and their mixtures, as indicated in the legend (proportions are given as v:v). (**A**) HeLa pLuc 705 cells were incubated for 24 h with solutions containing SCO alone (100 nM), nanoparticles of SCO and PF14 taken at MR 5 (PF14:SCO) with or without addition of 3 mM $CaCl_2$ or $MgCl_2$. (**B**) U87 MG-Luc2 cells were incubated for 48 h with solutions containing siRNA alone (15 nM), nanoparticles of siRNA and PF14 taken at MR 34 (PF14:siRNA) with or without addition of 3 mM $CaCl_2$ or $MgCl_2$. Viability of the cells was evaluated using WST-8 assay, and absorption of untreated cells was taken for 100%. Each dataset represents mean ± SD of at least one experiment, with each dot corresponding to one technical replicate. Data was analysed by one-way ANOVA with Dunnett's test. Asterisks indicate statistically significant difference, compared to untreated cells, * *p*-value < 0.05, ** *p*-value < 0.005, **** *p*-value < 0.0001. DMF – dimethylformamide, EtOH – ethanol, THF – tetrahydrofuran, MeOH – methanol, iPrOH – isopropanol, TMC – trimethylen carbonate.

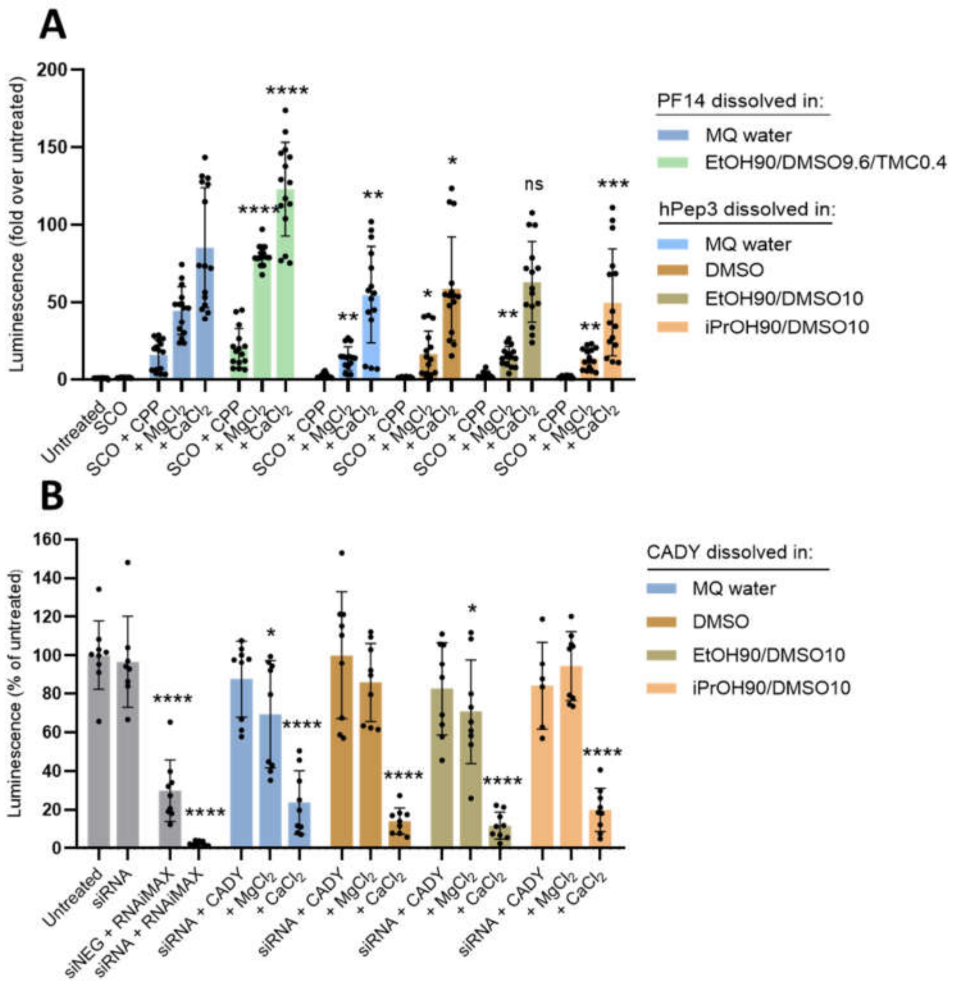

Figure S7. Effect of cosolvent on tranfection of SCO and siRNA by hPep3 and CADY. (**A**) HeLa pLuc 705 cells were incubated for 24 h with solutions containing SCO alone (100 nM), nanoparticles of SCO and PF14 or hPep3 [1] taken at MR5 with or without addition of 3 mM of $CaCl_2$ or $MgCl_2$. (**B**) U87 MG-Luc2 cells were incubated for 48 h with 17 nM siRNA, nanoparticles of siRNA and CADY [2] taken at MR34 with or without addition of 3 mM of $CaCl_2$ or $MgCl_2$. Each dataset represents as mean ± SD of at least three independent experiments, while each datapoint represents one technical replicate. Data was and analysed by one-way ANOVA with Tukey's (**A**) or Dunnett's (**B**) test. In the panel (**A**), asterisks show statistically significant differences, compared with the analogous solution from „PF14 dissolved in MQ water" group, and in the panel (**B**), compared with untreated cells, * p-value < 0.05, ** p-value < 0.005, *** p-value < 0.0005, **** p-value < 0.0001, ns – not significant.

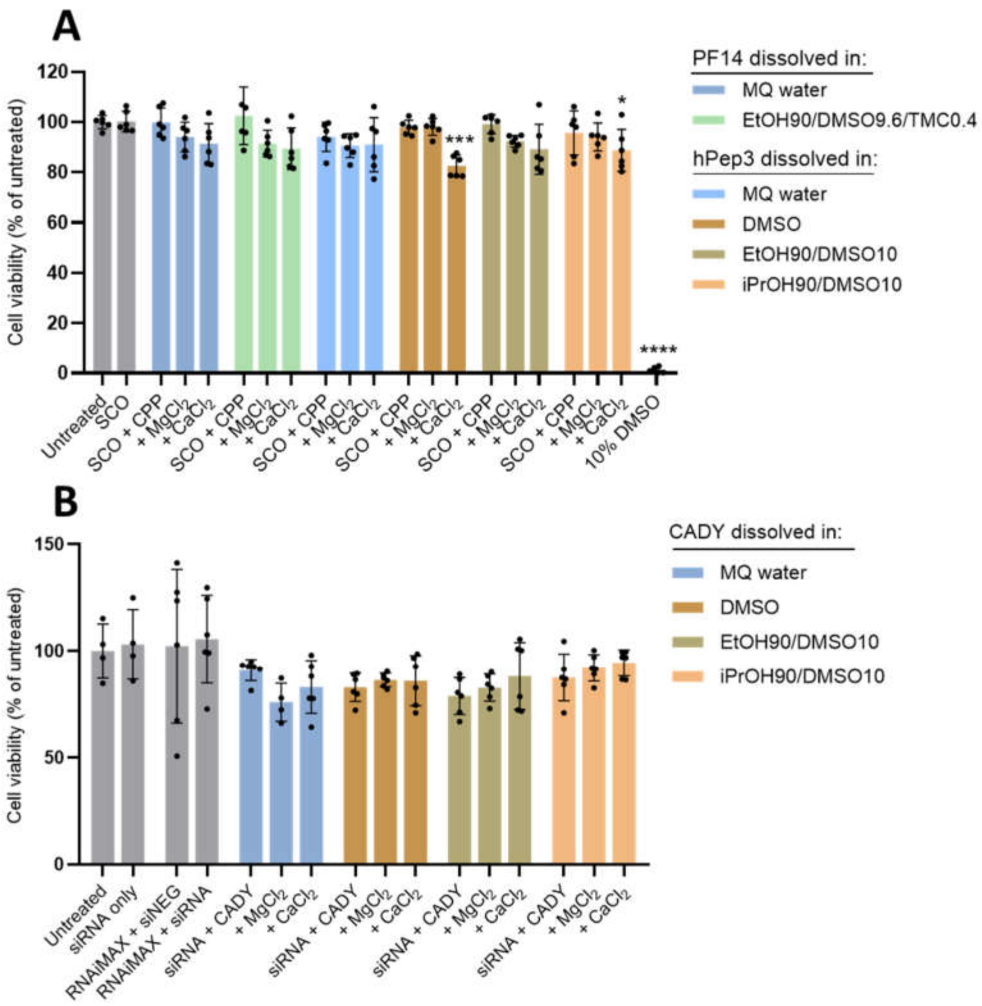

Figure S8. Effect of nanoparticles prepared of SCO and hPep3 (**A**) or siRNA and CADY (**B**), on viability of HeLa pLuc 705 or U87 MG-Luc2 cells, respectively. (**A**) HeLa pLuc 705 cells were incubated for 24 h with solutions containing SCO alone (100 nM), nanoparticles of SCO and PF14 or hPep3 taken at MR5 with or without addition of 3 mM of $CaCl_2$ or $MgCl_2$. (**B**) U87 MG-Luc2 cells were incubated for 48 h with 17 nM siRNA, nanoparticles of siRNA and CADY taken at MR34 with or without addition of 3 mM of $CaCl_2$ or $MgCl_2$. Each dataset represents as mean ± SD of two independent experiments. Data was analysed by one-way ANOVA with post-hoc Dunnett's test. Statistically significant differences compared to untreated cells are shown with asterisks, * p-value < 0.05, *** p-value < 0.0005, **** p-value < 0.0001.

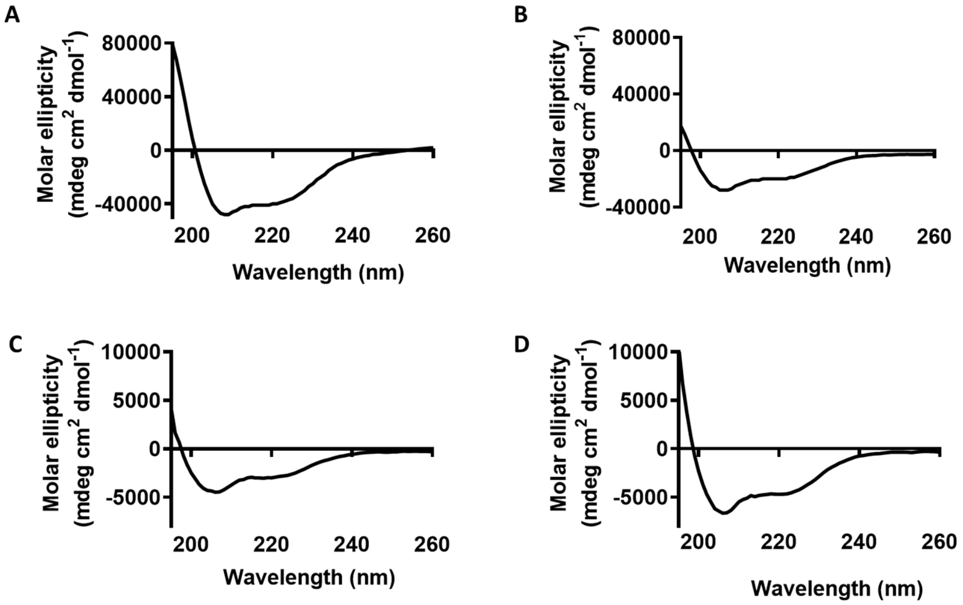

Figure S9. Circular dichroism (CD) spectra reflecting the secondary structure of PF14 dissolved in MQ water (**A**), EtOH (**B**), EtOH90/DMSO10 (**C**), and in EtOH90/DMSO9.6/TMC0.4 (**D**) after dilution into MQ water. Each 1 mM PF14 stock solution was diluted to 60-80 µM concentration in MQ water for the measurement.

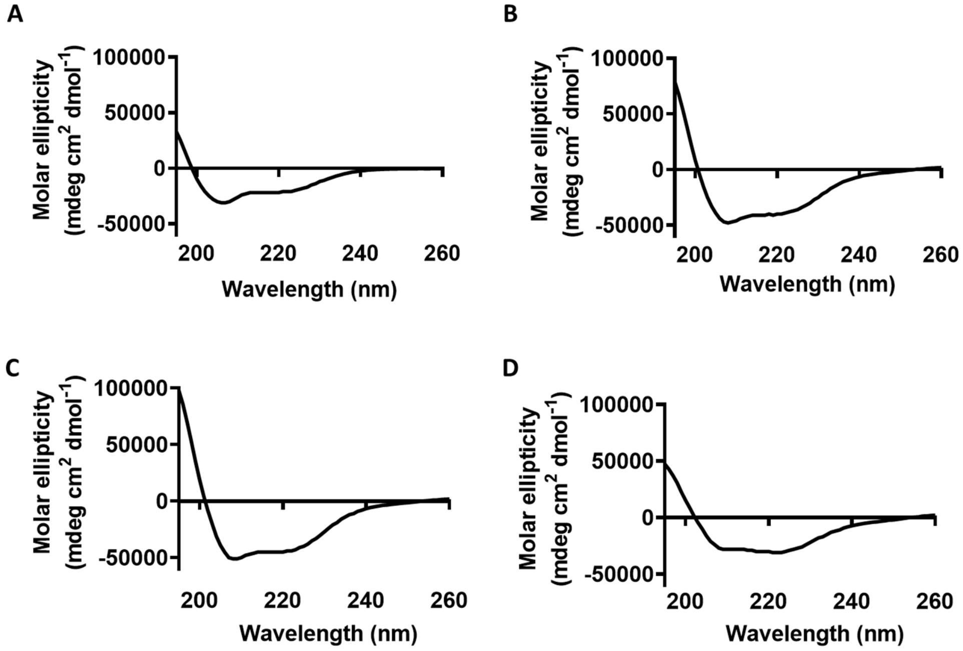

Figure S10. Circular dichroism (CD) spectra reflecting the secondary structure of PF14 alone (**A**); complex of PF14 with SCO (**B**); complex of PF14 with SCO and 3 mM $CaCl_2$ (**C**); and complex of PF14 with SCO and 3 mM $MgCl_2$ (**D**). PF14 was dissolved in EtOH90/DMSO9.6/TMC0.4 and each solution was diluted to 60-80 µM concentration of peptide in MQ water for the measurement.

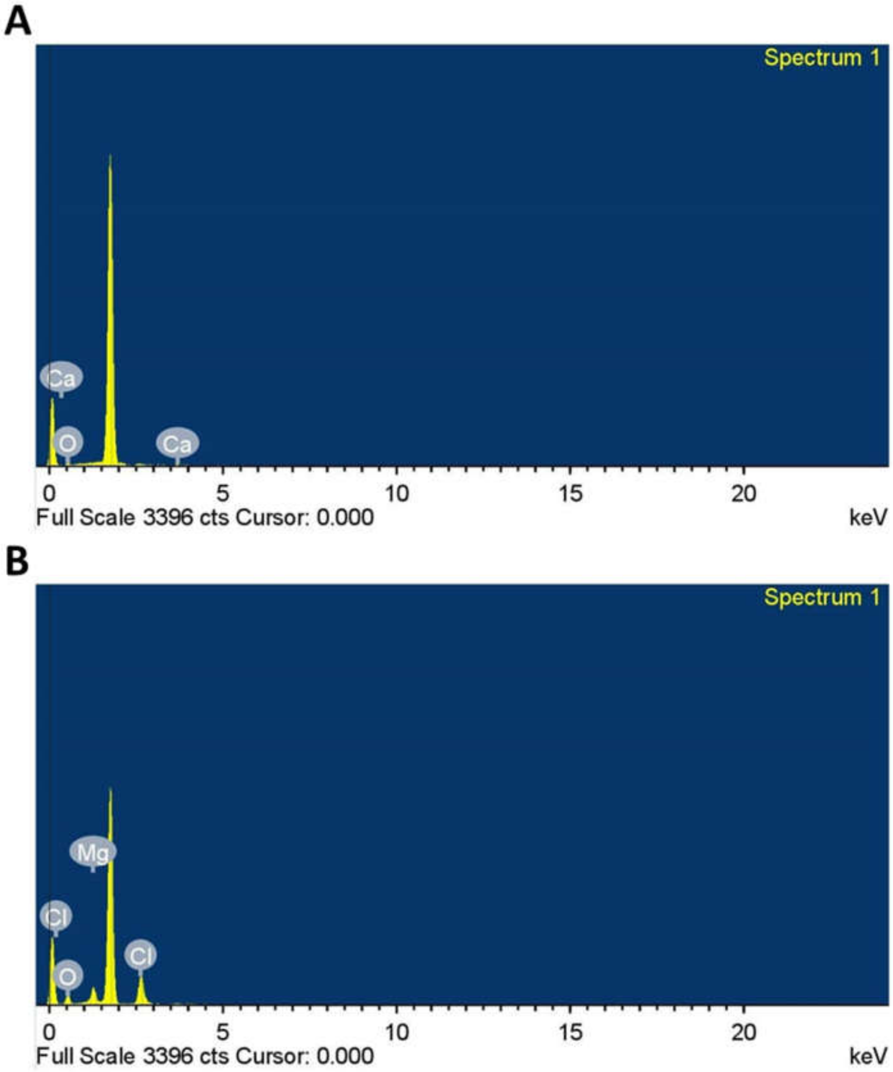

Figure S11. EDX spectra confirming the presence of Ca^{2+} (**A**) and Mg^{2+} (**B**) ions in nanoparticles assembled from PF14, SCO and $CaCl_2$ or $MgCl_2$, respectively.

References

1. Bazaz, S.; Lehto, T.; Tops, R.; Gissberg, O.; Gupta, D.; Bestas, B.; Bost, J.; Wiklander, O.P.B.; Sork, H.; Zaghloul, E.M.; et al. Novel Orthogonally Hydrocarbon-Modified Cell-Penetrating Peptide Nanoparticles Mediate Efficient Delivery of Splice-Switching Antisense Oligonucleotides In Vitro and In Vivo. *Biomedicines* **2021**, *9*, 1046. https://doi.org/10.3390/biomedicines9081046.
2. Crombez, L.; Aldrian-Herrada, G.; Konate, K.; Nguyen, Q.N.; McMaster, G.K.; Brasseur, R.; Heitz, F.; Divita, G. A New Potent Secondary Amphipathic Cell–Penetrating Peptide for SiRNA Delivery Into Mammalian Cells. *Mol. Ther.* **2009**, *17*, 95–103. https://doi.org/10.1038/mt.2008.215.
